# A Reaction-Diffusion Model of Cholinergic Retinal Waves

**DOI:** 10.1371/journal.pcbi.1003953

**Published:** 2014-12-04

**Authors:** Benjamin Lansdell, Kevin Ford, J. Nathan Kutz

**Affiliations:** 1 Department of Applied Mathematics, University of Washington, Seattle, Washington, United States of America; 2 Department of Biochemistry and Biophysics, University of California San Francisco, San Francisco, California, United States of America; Indiana University, United States of America

## Abstract

Prior to receiving visual stimuli, spontaneous, correlated activity in the retina, called retinal waves, drives activity-dependent developmental programs. Early-stage waves mediated by acetylcholine (ACh) manifest as slow, spreading bursts of action potentials. They are believed to be initiated by the spontaneous firing of Starburst Amacrine Cells (SACs), whose dense, recurrent connectivity then propagates this activity laterally. Their inter-wave interval and shifting wave boundaries are the result of the slow after-hyperpolarization of the SACs creating an evolving mosaic of recruitable and refractory cells, which can and cannot participate in waves, respectively. Recent evidence suggests that cholinergic waves may be modulated by the extracellular concentration of ACh. Here, we construct a simplified, biophysically consistent, reaction-diffusion model of cholinergic retinal waves capable of recapitulating wave dynamics observed in mice retina recordings. The dense, recurrent connectivity of SACs is modeled through local, excitatory coupling occurring via the volume release and diffusion of ACh. In addition to simulation, we are thus able to use non-linear wave theory to connect wave features to underlying physiological parameters, making the model useful in determining appropriate pharmacological manipulations to experimentally produce waves of a prescribed spatiotemporal character. The model is used to determine how ACh mediated connectivity may modulate wave activity, and how parameters such as the spontaneous activation rate and sAHP refractory period contribute to critical wave size variability.

## Author Summary

Both within the visual system and more generally, two general processes describe nervous system development: first, genetically determined cues provide a coarse layout of cells and connections and second, neuronal activity removes unwanted cells and refines connections. This activity occurs not just through external stimulation, but also through correlated, spontaneously generated bursts of action potentials occurring in hyper-excitable regions of the developing nervous system prior to external stimulation. Spontaneous activity has been implicated in the maturation of many neural circuits, however exactly which features are important for this purpose is largely unknown. In order to help address this question we construct a mathematical model to understand the spatiotemporal patterns of spontaneously driven activity in the developing retina. This activity is known as retinal waves. We describe a simplified, biophysically consistent, reaction-diffusion model of cholinergic retinal waves capable of recapitulating wave dynamics observed in mice retina recordings. This novel reaction-diffusion formulation allows us to connect wave features to underlying physiological parameters. In particular this approach is used to determine which features of the system are responsible for wave propagation and for the spatiotemporal patterns of propagating waves observed in both mice and other species.

## Introduction

Throughout the nervous system, correlated spontaneous activity drives developmental programs [1], [2]. Within the retina, these events manifest as slow, spreading waves of depolarizations and are thus termed retinal waves. Waves are observed in a variety of species and progress through three stages of development [3], [4]. They have been implicated in numerous developmental processes, including retinotopic map refinement [5] and the eye-specific segregation of retinal projections into layers of the thalamus [6]. In mammals, waves mediated by acetylcholine (cholinergic waves, or stage II waves) are the best characterized. They exhibit a slow wavefront velocity, random initiation site and direction of propagation, an interwave interval (IWI) which lasts tens of seconds, and constantly shifting wave boundaries. Precisely determining their role in development requires a controlled manipulation of these properties, which in turn requires a sound theoretical understanding of the mechanisms responsible for their generation. However, the complexity of their dynamics means that determining the connection between the generation and resulting spatiotemporal patterns of wave activity and underlying physiology is by no means intuitive and is reliant on computational modeling. This paper develops a biophysically consistent, yet simplified, conductance based model of the developing retina that is able to produce physiological waves, in order to elucidate this connection.

Retinal waves are believed to be initiated by spontaneous depolarizations of Starburst amacrine cells (SACs) whose processes reside in the inner plexiform layer (IPL) of the retina. In mice these spontaneous depolarizations are sparse: they occur roughly once every 15 minutes per SAC [7], where as in rabbit the rate is closer to once every 30 seconds. Following initiation, the dense, recurrent connectivity of the SAC network laterally propagates activity through cholinergic synapses [8]. After depolarizing, SACs exhibit a slow after-hyperpolarization current due to a cyclic-AMP sensitive, calcium-activated potassium channel [7], [9], which persists for tens of seconds. Consequently, waves propagate over only a finite domain of the retina, with their boundaries determined by regions still refractory from previous wave activity. A number of computational studies have tested this wave generation hypothesis, notably the models by Godfrey *et al*. 2007 [10], Hennig *et al*. 2009 [11] and subsequent study by Ford *et al*. 2012 [7] (refer to the reviews Gjorgjieva and Eglen 2011 [12] and Godfrey and Eglen 2009 [13] for more information on previous computational studies).

A striking feature of retinal waves is their variety of sizes, speeds and shapes, which differs from the more stereotyped behavior of spontaneous activity in other developing brain regions, for example in neocortex [14], [15]. Despite numerous theoretical studies, it is not clear what properties of the recurrent developing SAC network most contribute to these spatiotemporal patterns. Indeed, retinal waves sizes appear to be distributed according to a power-law [11], analogous to other examples of spontaneous activity in the nervous system. In cortex, such activity has been extensively studied as an example of a critical state phase-transition [16]. Additionally, diffuse release of ACh has been detected at the inner limiting membrane coincident with wave activity [7], suggesting that, like later stage waves mediated by glutamate [17], extra-synaptic diffusion may play a role in cholinergic wave propagation, though this has not been tested theoretically or experimentally.

In this work, we develop a theoretical, reaction-diffusion framework that integrates the key biophysical processes, including increased excitation due to acetylcholine diffusion and the slow after hyper-polarization of the SACs, to characterize spontaneous wave dynamics in the developing retina. This provides a framework to address the above questions of critical dynamics and mechanisms of wave generation and lateral propagation. The model is consistent with the above described mechanisms, but its purpose is to be as simple as possible while still being able to capture the defining spatiotemporal properties of retinal waves. We demonstrate the model is capable of producing physiological waves, as observed in mice [7], after which we address two questions. First, what are the conditions for spontaneous activity in the amacrine cell layer to propagate laterally as waves? We derive necessary conditions for propagating activity using techniques from non-linear dynamics that are made available through the use of a reaction-diffusion model. These conditions are shown to be consistent with known pharmacological and genetic manipulations. Second, once conditions for propagation are met, what are the conditions required for the spatiotemporal patterns of retinal wave activity to take the form of avalanches, as observed in multielectrode array recordings of stage II waves in a variety of species [11]? This is answered by appeal to a model of critical behaviour in a canonical model of forest fires.

## Results

### A physiological reaction-diffusion model of cholinergic retinal waves

**Figure 1:**
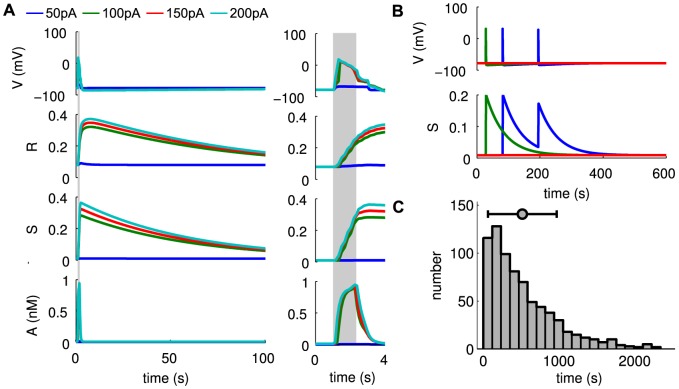
Isolated SAC dynamics. Starburst amacrine cells obey Morris-Lecar dynamics with voltage term 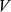, refractory variable 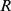, sAHP variable 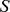 and acetylcholine concentration 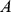. **A.** Time course of individual SAC dynamics following current injections of indicated size, injected at 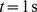 for 1.5 s (shown in grey). Model SACs' refractory period shows dependence on amount of current injected. **B.** Single cell dynamics showing sparse spontaneous depolarizations. Different colors represent different SACs. **C.** Distribution of inter-event intervals in stochastic simulation of isolated SAC. A threshold of −50 mV was employed. Bar plot shows mean and standard deviation.

As in previous models [7], [11], individual SACs are modeled according to Morris-Lecar dynamics [18], with an additional sAHP current activated by depolarization and subsequent calcium influx. The sAHP current generates recovery times on the order of a minute. Each cell is assigned the same recovery time scale, and the dynamics are such that recovery is minimally activity-dependent (Fig. 1A). Previous theoretical models [10], [11] and experimental observations [9] show that larger depolarizations increase sAHP duration. The lack of a strongly activity-dependent refractory period in our model does not affect the its ability to generate physiological waves. A noisy, excitatory current induces spontaneous depolarizations (Fig. 1B) at an average rate of once every 15 minutes per isolated cell, consistent with recordings in mice (Fig. 1C).

The dense synaptic connectivity of the SAC network and the potential role for extra-synaptic transmission suggests that a model based on local coupling between cells is appropriate. As such, coupling between amacrine cells is modeled via the volume release and diffusion of acetylcholine (ACh), and an excitatory post-synaptic current dependent on the local concentration of ACh. By taking a continuum limit of this amacrine cell network, a reaction-diffusion model is thus described (Methods). By modelling lateral excitation as a diffusion process the model is mathematically tractable. It should be emphasized that the diffusion of ACh is best thought of as an effective diffusion process, representing the combination of synaptic and extra-synaptic excitation through acetylcholine. Below we will demonstrate the inclusion of long-range connections, in addition to local, ‘diffusive’, ones has minimal impact in simulations.

The model is described in more detail in the Methods, but its notation is established here. For a SAC located at 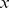, its membrane potential at time 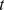 is described by 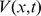. Three dynamic variables regulate the membrane potential: 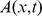, the extra-cellular ACh concentration which provides an excitatory current to the SAC; 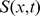, a calcium-dependent potassium channel which provides an inhibitory, slow after hyperpolarization (sAHP) current; and 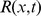, an inhibitory potassium channel modeled as in the original Morris-Lecar model. The vector 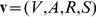
(1)then specifies the state of the system and obeys the following dynamics 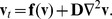



The matrix 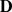 is a diagonal matrix whose specified entries indicate the diffusion coefficient of each dynamic variable 
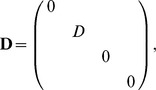
and whose blank entries represent zeros. The function 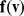 specifies the cell-intrinsic dynamics.

**Figure 2:**
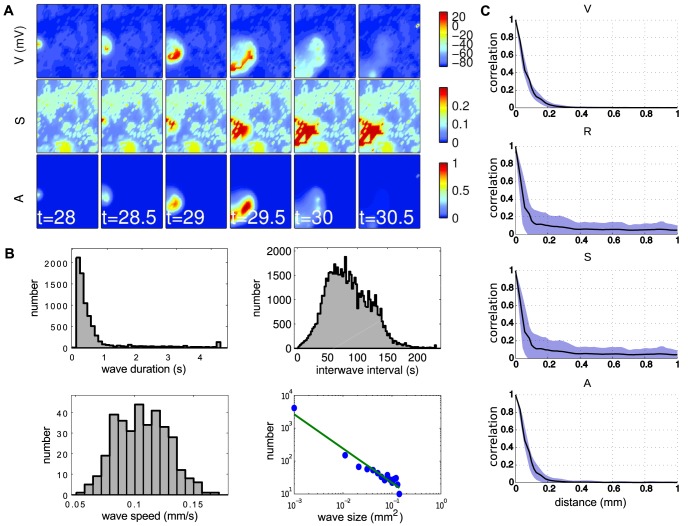
Model produces realistic cholinergic waves. **A.** Network dynamics showing spatiotemporal patterns of retinal waves **B.** Distribution of wave sizes, speeds, durations and inter-wave intervals from 2500 s of simulation. Mean wave size is 0.017 mm
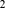
(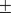
0.059 mm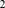), mean wave speed is 0.11 mm/s (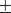
0.022 mm/s), mean wave duration is 0.63 s (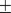
0.90 s), and mean inter-wave interval is 49 s (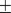
25 s). **C.** SACs exhibit variable participation in waves. Pearson correlation coefficient between a cell in the center of the domain and all other cells. The correlation coefficient for each variable is plotted as a function of euclidean distance between cells. Computed using one 2500 s simulation, with activity recorded every 0.1 s. Solid curve represents a loess moving average estimate of mean correlation as a function of distance. Shaded region highlights all points within one standard deviation of this mean curve.

Simulations show qualitatively that the model produces realistic waves (Fig. 2A; Movie S1). Waves propagate without bias in their initiation region or direction (due to the translational and rotational symmetry of the equations), occur on average once every 60 s, propagate at an average speed of 150 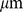 per second, and exhibit a broad distribution of wave sizes, all of which are consistent with *in vitro* recordings of mice retinal waves [7] (Fig. 2B). The uniform distribution of initiation points is expected given the homogeneity of the connectivities (diffusion coefficient) of the model. Some studies show that both *in vivo* and *in vitro* recordings contain a directional bias in propagation [19], [20], which could be modelled with a drift-diffusion model. However, since there is presently no physiological model for how this directionality occurs, we do not attempt to address these issues here.

The spontaneous firing of each cell is modeled as a Poisson process, which makes it simple to fix the per-cell spontaneous activation rate to be consistent with the recordings of Ford *et al*. 2012 [7]. The mean firing rate is set to be low, such that adjacent spontaneous activations are exceedingly rare, and hence waves are the result of a single SAC depolarization. However, the mean rate is sufficiently high to desynchronize the network, such that the correlation of activity of neighboring SACs quickly decays as a function of distance (Fig. 2C). Indeed, the correlation of both refractory variables (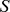 and 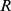) decays faster than either the voltage or acetylcholine variables, indicating neighboring SACs can exist in different refractory states and thus exhibit variable participation in waves [7], [11]. These features are consistent with physiological waves observed in mice. Further, by assuming a continuum model, the dimensionality of the dynamics can be drastically reduced, when compared with a network model. This feature is exploited in the following section.

### Necessary conditions for wave propagation

Having established the model produces realistic waves, in this section we determine under what conditions propagating activity exists. For this note that previous studies predict that the evolving mosaic of retinal wave activity is a result of the refractory period of SACs, which delineates shifting boundaries of future wave activity [7]. If previous wave activity is indeed responsible for the finite spatial extent of retinal waves then, were the amacrine cell network in a homogeneously recovered state, activity would spread across the entire retina without dissipation. Under this hypothesis, a prerequisite for the amacrine cell network supporting propagating retinal wave activity is thus that it should support traveling wave solutions were it provided an infinite spatial domain at rest – or that it should be an excitable medium.

To investigate parameters in which our retinal waves model is excitable is this sense, the amacrine cell network is studied as a non-linear reaction-diffusion system, in which we seek parameters under which traveling wave solutions can be constructed. The method is outlined briefly here, and described in further detail in Methods. We take advantage of the fact that the voltage and acetylcholine variables change on a faster timescale than either refractory variables. Following a singular-perturbation analysis outlined by Keener and Sneyd, 2001 [21], (treated in a number of other texts also, *e.g.* Ermentrout and Terman 2010 [22]), the dynamics can thus be broken into a fast and slow system:
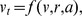


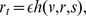


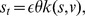


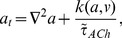
(2)where the lower-case variables denote dimensionless quantities corresponding to their upper-case equivalents of Equation 1. Singular perturbation analysis is used to study systems for which dynamics on more than one timescale need to be considered. For such systems, attempting to find approximate solutions by neglecting very fast or very slow components would result in an incorrect description of the dynamics. Here, the parameter 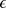 represents the ratio of the fast and slow timescales and is to be considered small (for the default parameters used in simulations 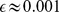). The model requires singular perturbation techniques because the small parameter affects the leading order derivative terms. The fast-system models the dynamics during the jump from the rest state to the excited (depolarized) state, or from the excited state to the refractory state. In one spatial dimension 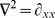, and the zeroth-order (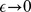) dynamics are therefore 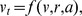


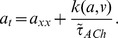



**Figure 3:**
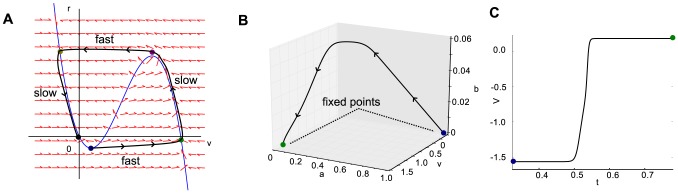
Construction of traveling wave-front. **A.** Fast-slow dynamics in the canonical Fitzhugh-Nagumo model of action potential generation. Black curve represents a trajectory of an action potential through phase space, in which a fast transition occurs between the rest (blue dot) and excited state (green dot), followed by slow excited dynamics (green to purple dot), another fast transition between the excited and refractory state (purple to yellow), and slow dynamics while refractory (yellow to black). Red arrows represent flow lines, and the blue curve is the 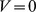 nullcline which defines the slow manifold (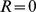 nullcline not drawn for clarity). **B.** The fast system here is described by three dynamical variables (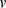, 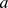, and 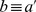). Shown here is the trajectory connecting the rest (blue) and excited (green) fixed points, defining the wavefront. **C.** Temporal voltage dynamics of the wave front.

In a moving frame with speed 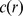, stationary solutions which connect the rest and excited fixed points are *heteroclinic* orbits which represent traveling front solutions. The basic idea of the wave front construction is outlined in Fig. 3.

**Figure 4:**
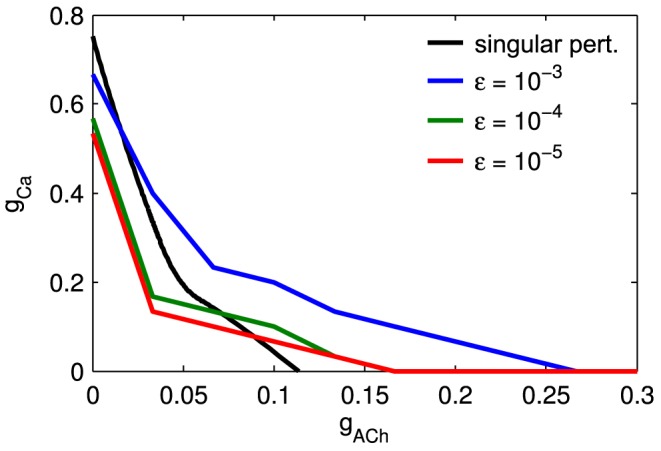
Parameter regimes which produce propagating activity. Numerical determination of retinal wave excitability threshold for different timescales 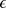 and excitability threshold determined through singular perturbation analysis, both as functions of 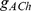, 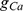. Each point on each curve indicates a point in parameter space in which the wavefront transitions from propagating to receding. Points in parameter space below each curve are therefore not excitable, while those above are excitable.

Parameters for which a heteroclinic orbit exists for a positive speed 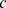 are parameters which support propagating activity. Parameters at the transition between a propagating, positive speed, traveling front and a receding, negative speed, traveling front (that is, when 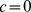) represent an excitability threshold. Fig. 4 demonstrates this excitability threshold over a two dimensional parameter space, computed using the above framework and, for comparison, computed using numerical simulations of the model for different values of 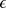 (the separation of fast and slow time scales). The analysis and simulations exhibit the same general behavior. The analysis method presented here is a more direct method for determining wave propagation because it is not necessary to set up and solve numerically a solution for each point in parameter space to be queried. Refer to Methods for more detail on the numerical simulations.

Both analysis and simulation demonstrate that excitability is sensitive to the value of both maximal conductances 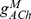 and 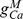. Further, excitability is independent of all aspects of the model not involved in the fast dynamics. This means that neither the spontaneous activation rate nor the duration of either refractory variable have an effect on wave propagation. Indeed, this is consistent with data presented in Ford *et al*. 2012 [7], which shows manipulations affecting the strength of the sAHP current have minimal effect on measured wave speed. In the sense defined here, excitability is also independent of the diffusion coefficient 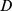, as it is scaled out of the dimensionless equations used to compute the excitability thresholds. Thus, beyond assuming a non-zero coefficient, the diffusion rate merely determines the speed at which waves propagate. The existence of spontaneous, propagating activity in the developing retina is thus determined by factors regulating individual SACs excitability and their synaptic strength.

**Figure 5:**
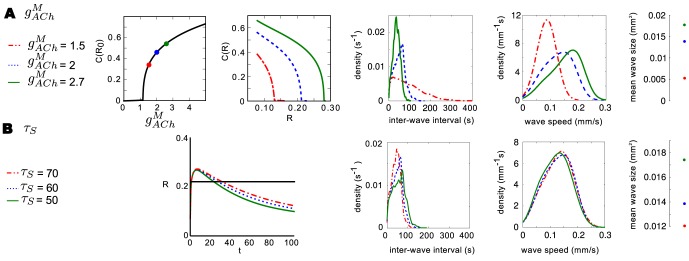
Modeling biophysical manipulations. **A.** Synaptic connection strength 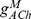 is varied. Sub plots from left to right: speed of wave front 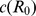 at rest (when 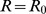) as a function of conductance 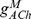, velocity indicates maximum wave-front speed since 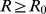 and 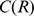 is monotonically decreasing, point at which 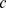 becomes zero represents excitability threshold; wave-front speed as function of refractory variable 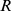 for three different values of 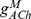; from 5000 s of simulation of model with indicated values of 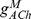 interwave-inteval; wave speed distribution; and mean wave size. **B.** Sub plots from left to right: dynamics of refractory variable 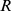 of individual SAC following depolarization with different sAHP timescales 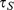, black line indicates refractory value above which 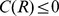 and thus represents an absolute refractory time period in which SAC is not sufficiently excitable to participate in future wave activity; from 5000 s of simulation of model with indicated values of 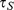
inter-wave interval; wave speed distribution; and mean wave size.

It is important to understand that this analysis is only for the case of a domain uniformly at rest. Of course, we would like to consider the existence of waves within a medium that is not uniformly recovered but for which some of the domain may be refractory from previous activity. Our analysis provides a necessary condition for the existence of propagating activity in this more general case: a network incapable of supporting wave propagation at rest is a network incapable of supporting wave activity when some of its cells are refractory. In this more general case, waves propagate not only if SACs are sufficiently excitable, but also if neighboring SACs are sufficiently recovered from prior depolarizations. The wave speed is then modulated by the refractory state as well (*e.g.* Fig. 5).

### Waves under physiological manipulations

The pharmacological or genetic manipulation of retinal waves forms a major component of their experimental study. In this section we show how two such common manipulations are recapitulated by our model and use the above analysis to interpret the effects of these manipulations. Using a biophysically inspired model is beneficial since model parameters have clear, experimentally determinable values.

#### Synaptic connection strength

We first investigate the effect of nicotinic acetylcholine receptor (nAChR) agonists/antagonists by varying the maximal synaptic conductance 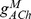 (see Methods for definition). Our analysis shows both the wave speed at rest and the wave speed as a function of refractory state 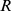 are highly sensitive to changes in 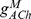 (Fig. 5A). A 25% reduction in 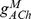 lowers the 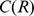 curve significantly, making the medium less excitable and waves more easily blocked by encounters with refractory cells. This manifests in simulations as a reduction in wave size, and a change in the inter-wave interval: from sharply peaked at a mean of approximately 50 s to a monotonically decaying function similar to the individual SAC spontaneous firing rate (Fig. 1), indicating that wave activity is localized and activations are primarily caused by spontaneous activity, not wave activity. This is indeed observed in pharmacological studies of mice, chick and turtle retina [23]–[25], which produce both a decrease in wave frequency and a decrease in wave size following treatment with nAChR antagonists.

Additionally, a study by Xu *et al*. 2011 [6] used transgenic mice in which only about half of SACs express functional nicotinic acetylcholine receptors, which reduces the effective coupling of the network. In these mice, wave frequency and speed were unchanged, whereas wave size was significantly reduced, as the analysis of our model predicts.

Conversely, a 25% increase in 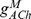
results in an increase in wave speed both from rest and as a function of refractory state, indicating that wave activity is more robust and less likely to be blocked by encounters with refractory cells. Indeed, in simulations this results in an increase in wave size and a more frequent and sharply peaked inter-wave interval distribution.

#### Modulation of sAHP current

We also investigate the effect on waves from varying the sAHP current. Experimentally, this is achieved through stimulating the cAMP second-messenger pathway via forskolin. We study this effect by varying the sAHP timescale 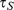. Since 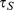 does not effect the fast time scale dynamics, our singular perturbation analysis does *not* provide insight into how 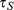 effects wave speed or propagation. However, using the model's excitability criteria does allow us to compute the duration of an absolute refractory period, which is the length of the period in which activation, either spontaneous or through incident wave activity, results in subsequent a refractory state that does not support a propagating wave. This period is shown to be on the order of 30 seconds, in accordance with experimental findings [25]. (Fig. 5B)

Retinal wave simulation *does* show how 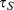 affects wave properties. An increase (respectively decrease) in 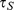 results in an increase (respectively decrease) in the interwave interval and a minimal change in wave size and wave speed (Fig. 5B). The reduction in IWI is expected given the decrease in absolute refractory period. The minimal change in wavespeed is also expected. Further, the moderate changes in 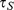 presented here should not affect significantly the wave size (larger changes in 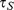 through which different wave behavior regimes are explored are investigated below). These results agree with the study of Ford *et al*. 2012 [7], which found that treatment with 1 *µ*M of forskolin resulted in roughly a 50% reduction in IWI, and only a moderate reduction in wave speed. The same increase in wave frequency and decrease in underlying sAHP current are also observed in ferrets and rabbit [9], [26].

Recently, Ford *et al*. 2013 [27] proposed that the sAHP current is established by the two-pore potassium channel TREK1. In this study TREK1 knockout mice are shown to exhibit retinal waves with a significantly reduced (approximately halved) interwave intervals. The present model, in which wave frequency is shown to be increased by a reduction of the timescale of sAHP recovery, is consistent with these findings.

It is possible the effect of forskolin is on the strength of the sAHP current, not on its decay rate, thus we also investigated the effect of changing the strength parameter 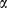, which determines how sensitive each SAC is to the slow refractory state 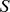. The effect was comparable to varying 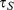 – a decrease in wave frequency and slight change in wave speed and size following an increase in 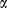 (Figure S3).

In summary, the preceding sections have demonstrated that our model produces physiological retinal waves, and that the existence of propagating activity can be understood by studying the model as an excitable medium. This allows for the effect of common genetic and pharmacological manipulations to be predicted.

### Spatio-temporal patterns of propagating activity

We now turn our attention to a second question: given parameters in which propagating activity exists, what model parameters determine their form? Indeed, the spatiotemporal patterns of cholinergic retinal waves are often similar, despite differences in a range of physiological parameters, in a range of species. How is this similarity of form maintained given variations in physiology? Given parameters in which propagating activity exists, this section addresses the nature of the resulting spatiotemporal patterns. Hennig *et al*. 2009 [11] notes that, both in MEA recordings and computer simulation, physiological waves take the form of avalanches, or power-law sized events. Thus it has been proposed that the developing amacrine cell network is in a critically configured state – a transition between a locally and globally connected network. Such neuronal avalanches have been observed and extensively studied in the both *in vitro* [28] and *in vivo* [16] cortex.

Here, we make an analogy between retinal waves and a model of self-organized criticality (SOC) [29], allowing us to determine for which parameters avalanches may be observed. A well-studied example of a complex system potentially demonstrating self-organized criticality is the Drossl-Schwabl forest fire model (DS-FFM) [30], [31], imagined as a grid in which each unit is either occupied (by a tree), ignited (burning tree), or empty (ash, burnt tree). At each discrete time step: 1) an occupied grid point spontaneously ignites with probability 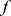, 2) burning trees ignite their occupied neighbors, 3) burning trees become empty and 4) empty sites regrow a tree with probability 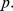 The analogy between the forest and the retina is clear: amacrine cells spontaneously fire at some rate and excite their nearest recruitable neighbors to also fire, after which these cells experience a slow recovery time, which proceeds at some average rate.

For a simulated lattice of 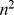 cells, representing 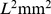 of retina (in our simulations 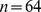 and 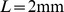), a simple heuristic derivation shows a relationship between 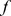 and 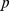 of the DS-FFM and the retinal wave model parameters: 
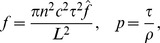
for wave speed 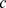, a per cell spontaneous firing rate 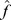, burst duration 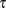, an effective refractory period 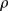, and length of domain 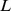. These values are determined either directly from model parameters (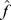, 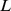, 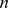), from the model analysis (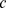 and 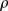), or from simulation (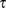).

The heuristic derivation is as follows: to estimate 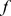 and 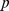 from retinal wave simulations, and relate it to the DS-FFM, we rescale time and space so that one time unit is the duration of a burst during wave activity (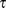); in this way after one time unit every active grid point is now refractory. And rescale space such that the area of one ‘lattice point’ is the area covered by all grid points excited as a result of one point spontaneously firing (initiating a wave) (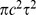); in this way, during one time unit, an active lattice point induces the nearest (and only the nearest) excitable lattice points to become active. When scaled in this way, the dynamics of our retinal wave simulations approximate the dynamics described by the rules of the DS-FFM. The probability of ‘regrowth’ (recovery) is 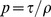, and the probability of a lattice point spontaneously firing is assumed to be the probability of a single simulated cell firing, multiplied by the number of cells that are included in that lattice point (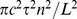).

In the DS-FFM, SOC is expected when [30]

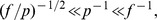
or, for the present model, when 
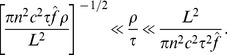
(3)


**Figure 6:**
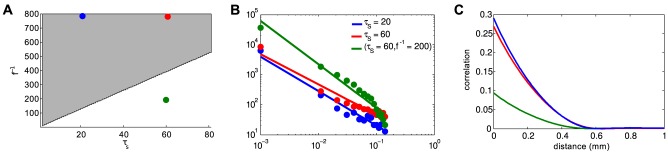
Power-law distributed wave-size retinal waves. **A.** Parameter space in which avalanches are expected (gray, Equation 3) and three sample points **B.** Wave size distributions (points) following 5000 s of simulation on a 128
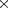
128 domain for specified values of 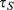 and 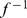. Solid lines represent log-linear least-squares lines of best fit, having slopes: 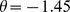 (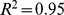, green), 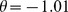 (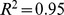, red) and 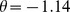 (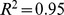, blue) **C.** Correlation in membrane potential between cells of a given distance apart.

In this regime, retinal wave, or forest fire, sizes are characterized by a power-law distribution with scaling exponent of approximately 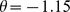 (simulation based [32], theoretical based [33], [34]). Parameters which have the largest and most direct impact on 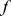 and 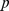 are the per cell spontaneous firing rate 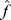, and the slow refractory variable 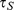 (refer to Fig. 5): these are the parameters which best determine when criticality may be observed. Fig. 6 demonstrates that, within the region described by Equation (3), wave sizes distributions approximately follow a power law with an estimated exponent close to the expected 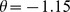. Conversely, simulations performed outside this parameter region are sub-critical and do not follow an approximate power-law. The same behaviour is observed when the distribution of wave duration (or lifetimes) are considered (Figure S4). This is further made clear by looking at the correlation in voltage activity between cells of a given distance from one another (Fig. 6C). Parameters for which power-laws are observed produce an initially high, but sharply decaying correlation function, while the sub-critical parameter set produces significantly less correlated activity. This is indicative of the smaller, more localized wave activity expected in a sub-critical system.

The relationship between the rate of spontaneous firing 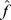, and the slow refractory variable 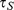, dictates whether the network exists in a critical state. This simple inverse relationship (Fig. 6) can explain how robust waves with similar spatiotemporal characteristics exist in very different parameter regimes. Experiments in mice indicate that SACs depolarize infrequently but exhibit sAHPs lasting as long as two minutes [7], whereas experiments in rabbit [9] show a spontaneous depolarization rate an order of mangitude higher and sAHPs that are substantially shorter. While these two systems have cellular parameters that differ on an order of magnitude, their collective network activity is very similar. This can be explained by the fact that the ratio of 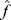 to 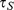 is conserved across species.

This analysis can thus be used to predict, given particular parameters for a particular species, whether or not the wave-size distribution will be critical or sub-critical. Further, since both parameters varied here, 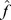 and 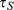, have no bearing on the excitability of the medium, the analysis of the spatio-temporal patterns of the amacrine cell network separates neatly into two stages. Firstly, if the medium is not excitable then only small, localized activity is expected. If the medium is excitable then, secondly, the spatio-temporal patterns expected will depend on the value of both 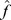 and 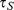, as discussed here.

## Discussion

### Comparison to previous studies

Close collaboration with experimentalists means computational modeling of retinal waves has been notably successful (refer to the reviews Gjorgjieva and Eglen 2011 [12] and Godfrey and Elgen 2009 [13]). Models have helped identify SACs as the cell layer which both initiates and laterally propagates waves [35], and have highlighted the importance of an activity-dependent refractory period to the generation of physiological waves [10]. Most recently, the models of Hennig *et al*. 2009 [11] and Ford *et al*. 2012 [7] (henceforth referred to by their first authors) have investigated how the cell-intrinsic spontaneous firing rate and the duration of each cell's refractory period contributes to physiological waves.

Godfrey and Hennig both propose the importance of a refractory period that scales with the degree of excitation. The Godfrey model was deterministic, and thus was reliant on the activity dependent refractory periods to make the dynamics chaotic and therefore consistent with observed waves – variable in size, and with shifting wave boundaries.

In the Hennig model, the competition between the synchronizing force of waves and the desynchronizing force of spontaneous activity is balanced at physiological wave parameters. This was adjusted in the Ford model to match the observed low spontaneous activation rate observed in mice SACs. Physiological waves were then only observed when each cell's refractory time scale was allowed to vary randomly (a hypothesis also pursued in Feller *et al*. 1997). Ford hypothesizes that this cell to cell variability is thus a necessary component of the developing amacrine cell network.

We find that neither hypothesis is necessary in producing realistic waves – our model does not contain an activity-dependent refractory variable or cell to cell variability in parameters. Our model is stochastic, consisting of sparse spontaneous depolarizations with enough strength to initiate a wave by themselves. These strong, sparse depolarizations are sufficient to induce variability in refractory periods. It remains to be shown how this might change in other species whose spontaneous depolarization rate is much higher, and in which cell-cell variability or activity-dependent refractory periods may play a more important role.

### Experimental validation of the model

The model makes several predictions. Firstly, it provides a way to systematically investigate the effect of different parameters on both wave propagation and spatiotemporal patterns. One way to confirm the model experimentally would then be to match (more closely than was able to be performed in the present study) the parameters of the model to observed wave statistics such that in a quantitative fashion the effect of particular pharmacological manipulations (*e.g.* nAChR antagonists) could be reliably reproduced. The test would then be to predict the outcome of a different pharmacological manipulation (*e.g.* cholinesterase inhibition) that the model was not calibrated against.

A simpler validation would be the following: a prediction the model makes is that certain parameters affect only the existence of propagating activity, while certain parameters affect only the form of that propagating activity (power-law distributed or otherwise). Thus manipulations that affect only parameters within one of these classes should have no effect on behaviours determined by parameters in the other class. For instance, the only effect changes to parameters of the sAHP current, according to our analysis, is on the form of the activity – spiral waves, large domain-covering waves, *etc* – not on their existence.

Finally, the model predicts the existence of power-law distributed activity within a particular parameter range based on an analogy to a forest fire model. There are many different models of self-organized critical systems which have slightly different rules and slightly different statistics, and it is not clear that the forest fire model indeed is the best analog. These different models exhibit different sub-sampling artifacts, when only a subset of cells are observed, and these effects thus provide a way of teasing apart different SOC candidates [36]. Examining sub-sampling effects in our model's simulations, and in high-density recordings of *in-vivo* or *in-vitro* retinal waves, would provide a way of determining which model exhibiting criticality is most appropriate.

### On the extra-synaptic diffusion of ACh

Previous retinal wave studies have hypothesized an extra-synaptic agent as responsible for wave propagation: Burgi *et al*. 1994 [37], for instance, noted that the relatively slow wave speed is consistent more with an extra-synaptic agent than either gap junction or direct synaptic stimulation, and proposed extracellular potassium as a source of lateral excitation. This particular hypothesis was later discredited. However diffuse action of ACh within the IPL has been observed coincident with wave activity, suggesting that the volume transmission of acetylcholine may instead play a role [7], [38]. Thus, although direct recurrent synaptic connections between SACs both in mice and in rabbit have been observed [7], [8], there is evidence that the diffuse release of ACh is responsible for wave propagation: synaptic currents recorded during waves last several seconds beyond depolarization, suggesting excitation via an extra-synaptic agent; and retinal waves drive ON and OFF RGCs [39] and cells in the inner nuclear layer [40], despite the restricted stratification of SAC processes within the IPL that do not form direct contacts with all cells, again suggesting propagation via volume transmission.

It is worth pointing out that extra-synaptic neurotransmission is observed throughout the developing nervous system to produce correlated spontaneous activity, and thus may be a general phenomenon regulating a diverse range of developmental programs. This may happen when the development of neurotransmitter release through synaptic vesicle fusion precedes the expression of the uptake transporters and breakdown enzymes required to prevent spillover into the extra-cellular space. For a the review of the role of extra-synaptic stimulation in generating spontaneous activity refer to Kerschensteiner 2013 [41].

The role of volume transmission has been explored in cortex [42], hippocampus [43], as well as extensively in stage III (glutamatergic) retinal waves [17], [44], [45]. In stage III waves extra-synaptic glutamate mediates lateral connections between bipolar cells.

The model presented here represents a theoretical validation that volume release of ACh is able to mediate stage II retinal waves. However, further experimental validation is needed beyond the above observations and this model. It should therefore be emphasized that the motivation of writing a model based on diffusion of acetylcholine is not to test the role of volume transmission. The dense lateral connectivity observed between SACs (synaptic or otherwise) means that lateral excitation is well approximated by a diffusion process – the model's diffusion of acetylcholine should be interpreted as a phenomenological, or effective, diffusion, not as a literal diffusion process based on the net Brownian motion of molecules which allows for singular-perturbation analysis to be performed.

Indeed, the extent of a SAC's arborization is only approximately 100 microns, so that even if synaptic terms were explicitly included their connectivity would be well-modelled by a short-range Gaussian weight function similar to the Gaussian kernel that results from modelling interactions as occurring through a diffusion process [46]. The only cells in the retina with long range connections are other types of GABAergic amacrine cells, which do not alter waves since GABA antagonists play a minor role in shaping the spatiotemporal features of waves. For these reasons models with long-range connectivity were not pursued in this study.

### Stage II retinal waves and development

Retinal waves are one example of spontaneous activity in the nervous system instructing developmental processes. A number of processes appear to rely on the activity generated by retinal waves. Indeed, they have been shown to coordinate activity throughout the visual system [19]. In animals in which waves are blocked, the formation of the retinotopic map [47], the separation of retinogeniculate projections into eye-specific layers of the LGN [6], and the formation of ocular dominance and orientation selectivity [5] (and references therein) are all adversely effected.

Retinal waves are thus believed to provide a naturalistic stimulus from which downstream connections can be refined and stablized. However, it remains unclear how important the spatiotemporal patterns of the activity are, or whether any activity, regardless of its properties (size, speed, typical shape), would suffice to provide input for correct development to occur. Hennig *et al*. 2009 [11], for instance, suggests that the critical form of the retinal waves are a useful feature, because, by definition, critical-state activity consists of events possessing no intrinsic length scale, they thereby provide an input without bias to any particular feature length. Godfrey *et al*. 2009 [48], on the other hand, investigates this question explicitly in a model of retinotopic map refinement and finds that the only feature which affects the degree of refinement is the spatial correlation activity function – the size, speed and frequency of the waves otherwise had no effect.

If correlation structure of retinal activity is the main factor regulating downstream developmental processes, then how is this structure dependent on the spatiotemporal properties of retinal waves? Are scale-free, critical ‘avalanches’ the only form of activity that provide the necessary correlation structure? In other areas (*i.e.* cortex), a network operating at a critical state can be shown to be optimal for information processing, and for maintaining a high dynamic range [49], [50]: there is some functional role for criticality (as discussed in Shew and Plenz 2013 [51]), but its significance remains controversial [52]. Retinal waves provide a useful study into the functional role of criticality, as they occur within well defined and understood circuitry where the above questions can be asked and thus critical activity can (potentially) be assigned a specific developmental role.

### Self-organized criticality

Our study focused in particular on a forest fire model extensively studied in the context of complex systems and critical phenomena for its potential demonstration of self-organized criticality (SOC). This connection between correlated activity within the central nervous system and the DS-FFM has been noted before (*e.g.* Buice and Cowan 2009 [53]). The DS-FFM model is not without its issues: while initial theoretical results [30], [33] provided justification for numerically observed scaling behavior, subsequent, more extensive, numerical analysis [32] demonstrate the power-law scaling behavior to be transient and not reflective of the ‘true’ asymptotic behavior. Indeed, a variety of scaling parameter (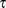) estimates have been observed for different parameter values and lattice sizes (refer to the summary presented in Pruessner 2012 [54]).

In theory, critical behavior is expected as the ratio 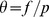 tends to infinity. However, this limit corresponds to lightning strikes becoming increasingly rare, such that in simulations, for sufficiently large 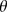, strikes are rare enough that the entire domain recovers by the time the next strike hits, and the resulting dynamics are simply that the fire spreads across the entire domain. Thus, in practice, critical behavior is to be expected when 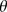 is large, yet the characteristic length scale remains small compared to the lattice size. In addition to this behavior, for sufficiently large 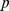 values spiral wave fronts are observed [55]. These behaviours – propagating ‘critical’, stationary rotating spirals and radially symmetric domain-covering waves are the three regimes of spatially extending activity observed in simulation of the present model, and as classified in previous models [56]. The latter two behaviours are likely to occur in parameter regimes where the spontaneous activation rate is sparse, thus creating large scale structures, and where, relative to the wave-front speed, the rate of recovery is either too fast (spiral waves) or too slow (domain-covering radial waves). Though more systematic study is needed in these cases, we argue the principle value of the analogy to the DS-FFM is not evidence of critical behavior in the developing retina (as it is not clear the DS-FFM is critical in a strict sense), but is the insight it provides into when and how these different regimes will be observed.

### Wider context

This work shows that a model based on lateral excitation through the diffusion of ACh can account for physiological retinal waves, and can predict how wave dynamics consequently depend on biophysical parameters. Specifically, by determining when the retinal network is excitable and through analogy to a canonical forest-fire model, the spatiotemporal patterns observed in different parameter regimes can be understood. This framework is shown to be consistent with previous experimental studies.

We note that the nature of this analysis is novel: the singular perturbation and traveling front construction analysis presented here has not been extended before, from the well studied and more stereotyped waves studied in cortex, to the considerably more complicated wave behavior observed in the developing retina.

A number of avenues for future work present themselves. First, the reaction-diffusion component of the model can be naturally applied to glutamatergic waves, for which there is more significant evidence that diffusion is indeed a key excitatory process. Mechanistic models of stage III waves have recently been developed [44], [45], and would benefit from computational modeling to establish their validity. It is proposed in stage III waves that lateral excitation occurs via the diffusion of extra-synaptic glutamate released by bipolar cells in the ganglion cell layer, and that GABAergic connections from amacrine cells to bipolar and ganglion cells provides a source of inhibition that is absent during stage II waves. Changes to the present model to a model of stage III waves would therefore be significant, however the framework to both analyze and simulate the resulting model would remain the same.

Second, the connection between between criticality and development can also be explored in much more depth than was performed in this study.

We believe the approaches developed here may also be applied to other forms of spontaneous and correlated activity in the nervous system.

## Models

### The model

SACs are assumed to obey dynamics based on a Morris-Lecar [11] model, which includes a quasi-stationary voltage dependent calcium conductance. The voltage dynamics are described by 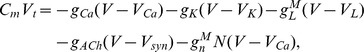
where 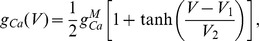


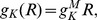


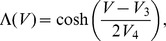


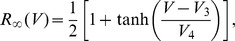
are the standard Morris-Lecar auxiliary functions. The model also includes an ACh conductance (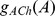) which depends on the local concentration of acetylcholine (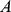): 
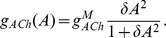



Additionally, a slow AHP variable (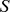) is activated according to a voltage-dependent function 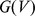 with time scale 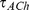: 
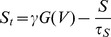


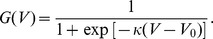



The sAHP variable enters the dynamics through its effect on refractory variable 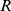 as follows 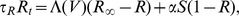
where 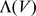 is defined above as part of the Morris-Lecar equations.

Cells are indexed by a continuous spatial parameter 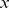 such that the state space is described by the tuple 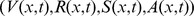, where 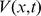 is the membrane potential. On spiking, cells release ACh at a voltage dependent rate 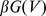 which diffuses with coefficient 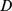:
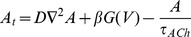


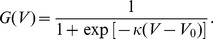



Coupling between SACs occurs only through the diffusion of ACh.

To induce spontaneous depolarizations, a noisy, excitatory current is included in simulations: 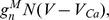
where at each time-step 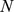 is a Bernoulli random variable (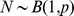). Refer to Table 1 for the parameter values used. The value of 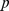 is chosen such that the mean waiting time between sponataneous noisy channel openings matches approximately the rate of intrinsic spontaneous bursts observed in mouse retina recordings [7].

**Table 1:** Parameters for retinal waves model.

Parameter	Value	Parameter	Value
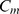	0.160 nF	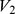	20 mV
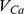	50 mV	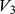	−25 mV
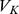	−90 mV	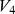	40 mV
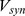	50 mV	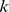	2
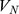	50 mV	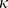	0.2 mV 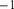
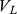	−70 mV	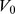	−40 mV
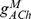	2 nS	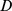	0.01 mm 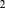 s 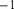
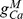	10 nS	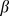	5 nM  s 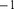
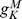	30 nS	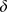	800 nM 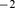
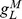	3 nS	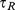	5 s
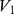	−20 mV	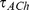	0.2 s
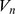	50mV	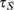	60 s
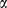	2	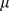	5
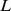	2 mm	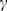	0.3 s 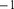

**Dimensioned parameters used in numerical simulations unless otherwise specified in text.** The chosen length of domain (2 mm) and a simulation of a 64
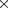
64 square lattice corresponds to approximation one SAC per grid point assuming an average distance between SACs of approximately 30 microns.

### Computational methods

Numerical integration was performed using a time-splitting method, in which the diffusion term was computed using a locally one dimensional Crank-Nicolson method and the reaction term was computed using a two-stage Runge-Kutta method. Simulations were computed in MATLAB (version 7.14.0; R2012a. Natick, Massachusetts: The Mathworks, Inc., 2012) using a 64×64 grid with a fixed step size of 1 ms. The stochastic conductance variable was updated every 10 ms. Simulation data used in statistical analysis was from 2500 s of simulation on a 4 mm^2^ domain (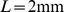), following a 500 s warm-up period. The dimensions chosen mean that one grid point has a length of approximately 30 *µ*m – roughly the density of SACs in the inner plexiform layer [56]. Simulations were performed on a dual 6-core Intel Xeon 3.07 Ghz machine with 24 GB RAM, running Ubuntu 12.04. MATLAB code to run simulations is available for download at: https://github.com/benlansdell/retinalwaves.

### Simulation statistics

Statistical analysis is performed as follows: snapshots of the simulation are taken every 10 time steps (10 ms). To remove boundary effects, grid points 5 units or less from the boundary are not considered for analysis. All grid points whose potential is above a threshold of −60 mV are labeled as active. All active points adjacent to one another are assigned a common wave number. For each set of cells assigned that number: the time from the first active cell to the last active cell is its wave duration, and the total number of cells is its size. The wave speed is calculated as in Blankenship *et al*. 2009 [17]: the wave initiation and termination points are identified and the path the wave front travels between these two points is computed. This allows the maximum wave front speed for that wave to be computed, and the distance this wave front travels divided by the wave duration gives an average wave speed. To give better estimates of the wave front speed, waves which involve the activation of fewer than 50 cells or whose duration is less than 1 s are ignored, and waves which do not have a distinct initiation point and are therefore the result of two waves colliding are also omitted (Figure S1). The interwave-intervals are computed by considering all above threshold wave activity, collisions or otherwise, and, for each grid point, measuring the time between successive threshold crossings. A minimum IWI of 2 s was imposed.

### A note on thresholds and smoothing voltages

Previous models of retinal waves consisted of two layers: an amacrine cell layer, which initiated and laterally propagated activity, and a ganglion cell layer, which acted as a smoothed ‘read out’ layer [35]. Subsequent models did away with the ganglion cell layer, and modeled only SACs [10], [11]. However, without this read-out later, wave analysis based directly on thresholding SAC potentials is noisy, making wave front tracking more difficult and wave ‘collisions’ more common. In order to compare simulated waves to recorded waves, Godfrey *et al*. 2007 [10] thus compute a calcium response variable, in which wave fronts are smoothed.

Our simulations (Fig. 2A), and experiments [7] show highly variable participation in wave activity on a cell-cell basis. Thus to compare our simulations to calcium imaging data, which is based on filtered RGC activity, we lower the threshold for which a cell is counted as participating in a wave to the ACh release threshold (near −55 mV), instead of its spiking threshold. This thus reflects the ‘general excitation’ of the region, not individual SAC activity, which makes it better suited for comparison with calcium imaging data. With this threshold, smoothing our data did not have a significant effect on wave labeling, so was not implemented (Figure S2).

### Bifurcations

AUTO [57] was used for the numerical continuation of traveling fronts and bifurcation analysis.

### Mathematical analysis

In order to analyze the model we first perform non-dimensionalisation, the parameters and scalings are outlined in Table 2. Non-dimensional dynamic variables are named in lower-case. To non-dimensionalise we make the following change of variables: 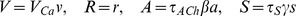
and scale time (dimensioned quantity here denoted by a capital 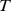) and space (capital 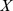) such that 
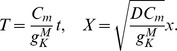



**Table 2:** Dimensionless parameters for retinal waves model.

Parameter	Relation	Value	Parameter	Relation	Value
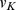	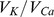	−1.8	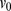	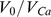	−0.8
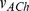	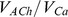	1	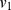	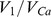	−0.4
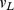	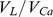	−1.4	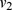	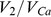	0.4
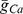	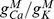	1/3	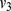	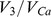	−0.5
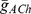	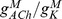	1/15	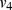	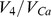	0.8
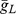	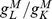	1/10	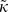	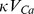	10
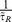	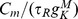	0.001067	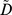	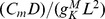	1.333 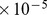
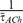	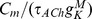	0.02667	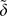	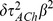	800
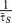	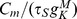	8.88 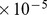	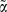	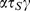	36

Dimensionless parameters used in analysis of model and their values.

Then:
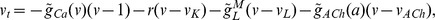


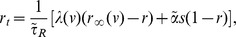


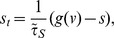


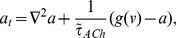
with
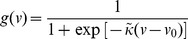


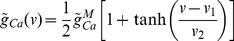


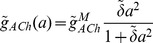


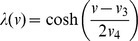


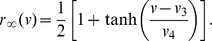



To proceed, let 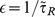, 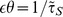 and 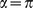


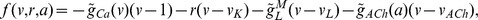


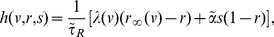


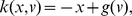
to give:
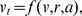


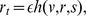


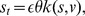


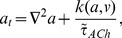
(4)which we analyze as a fast-slow system as is standard (refer to Ermentrout and Terman 2010 [22], or Keener and Sneyd 2001 [21]). The methods described below make no attempt to rigorously establish the existence of traveling fronts or waves.

### Wave fronts

This scaling of space and time is appropriate in regions where the diffusion term is relevant, thus the system obtained by letting 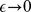


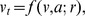


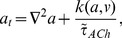
(5)provides an approximate description of the wave fronts and backs. The variable 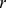 is here considered a parameter. To construct wave front solutions in one spatial dimension, change coordinates to a frame moving with speed 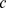: 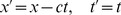
so that 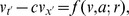


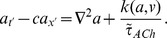



A traveling front corresponds to the stationary solutions:
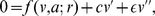


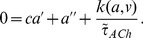
(6)where 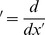. A bounded wave front solution, if it exists, corresponds to a *heteroclinic* orbit connecting the rest fixed point to the excited fixed point of Equation 6. In order to obtain this heteroclinic orbit, Equation 5 was simulated in MATLAB with a sigmoid function connecting the rest and excited fixed points for initial data. Once the solution had sufficient time to converge to the traveling front solution, its profile was saved, its wave speed calculated, and these two pieces of data put into the AUTO subpackage homcont to perform continuation on the system described in Equation 6. This allows for the computation of 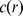 – the wave speed as a function of refractory state.

### Excitability thresholds

We seek to determine when the medium is sufficiently excitable to support a traveling front solution. In two variable neuronal systems such as Fitzhugh-Nagumo or Morris-Lecar, in which case the fast dynamics are one dimensional, an energy argument can provide conditions under which the medium is excitable (*e.g.* Ermentrout and Terman 2010 [22]). This is not possible since the fast dynamics are here two dimensional. So, let 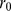 be the unique fixed point of Equation 4. We aim to find parameters for which 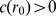, which, assuming the front we are studying is excited to the left and at rest to the right, will correspond to a propagating front. Parameters for which 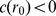 will generate a receding front, and thus parameters for which 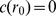 corresponds to the transition between excitable and not. This is found using AUTO.

These results are compared to model simulations without the noise channel. The simulations are performed for different parameters for a simulated time of 10 s, with an initial condition in which the domain is at rest besides a small cluster of grid points on one side of the domain. Excitability is determined by measuring if activity above an ‘excited’ threshold is observed on the other side of the domain – from which we infer that a wave must have traversed the simulated retina.

### Relation between one dimensional analysis and two dimensional simulations

Our analysis is performed in one spatial dimension only. Moving to two dimensions has the following effect: the Laplacian in radially symmetric polar coordinates is 
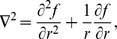
which, for large 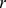, approximates the one dimensional Laplacian operator. Thus we expect, for waves of large radius, the results we find for one spatial dimension will apply. Indeed, performing the above simulations to determine excitability threshold with either a small cluster of initially excited cells (thus simulating a 2D, but radially symmetric solution) or with an entire strip of initially excited cells (thus simulating essentially the 1D dynamics) showed negligible difference (S5 Figure).

## Supporting Information

Figure S1 **Determining speed of wave front for two example waves.** Black corresponds to earlier times, and orange to later. At the latest time the wave is active, the wave front which connects the most distal point from the initiation point is tracked. Starting at this final point and moving backward in units of 0.5 s, the next closest active point to the current one draws the wave front trajectory in reverse, as demonstrated in **A.** This process does not make sense if a wave is the result of a collision, as shown in **B.** Waves that are involved in a collision have more than one start point (additional start point shown as single green circle), meaning there is ambiguity in how to apply the wave speed algorithm. The wave speeds for these waves (shown in right subplot) are not counted in our analysis.
(EPS)

Figure S2 **Effect of thresholds and smoothing on wave labeling.** Snapshot of a thresholded and subsequently labeled wave from voltage data **A.** without **B.** and with **C.** smoothing. Threshold was set to −60 mV to produce wave forms which, by eye, match waves shown in voltage data.
(EPS)

Figure S3
**Effect of varying sAHP sensitivity parameter** 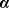 **.** Statistics following 5000 s of simulation of model with indicated values of 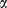 – interwave interval; wave speed distribution; and mean wave size. Comparison to Fig. 5 shows varying 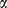 has a similar effect to varying the timescale 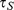. Note that varying the sAHP sensitivity parameter 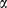 or varying the rate of activation parameter 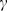 have identical impact on the model – as can be seen from the dimensional analysis summaryized in Table 2.(EPS)

Figure S4
**Distributions of wave duration (lifetime).** Distribution of wave duration on logarithmic scale following 5000 s simulation on a 128×128 grid point domain for specified values of 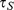 and 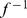. For reference, the dotted black curve shows a theoretical distribution of the form 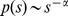 for 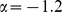 as observed in large-scale simulations of the DS-FFM model [54]. Comparison to Fig. 6 shows that SOC is not predicted for the parameters drawn in green, which indeed is clearly sub-critical.(EPS)

Figure S5
**Determination of excitability thresholds through numerical simulation, comparison between 1D and 2D simulations.** Curves show parameter values for which medium is excitable, estimated through numerical simulation of the model as described in ‘Models: Excitability thresholds’. Computations are performed with an initial condition of either an excited strip of SACs **A.**, or an excited point of SACs **B.**, thus simulating the 1D and 2D radially symmetric systems, respectively. Computed for three different values of time-scale parameter 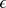. The figures are identical, indicating that the excitability thresholds obtained from analysis of the 1D system can also apply to a 2D domain.(EPS)

Movie S1
**500 s of simulation of retinal waves model.** Simulation of 4^2^ mm of retina played at 10× speed. Left panel to right panel: voltage potential (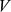), sAHP variable (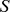), and acetylcholine variable (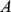). A higher resolution version can be found at https://github.com/benlansdell/retinalwaves.(MP4)
